# Supplementary material for: Mechanism of Plantamajoside in inhibiting ferroptosis of pancreatic β cells and treatment of T2DM via activation of the xCT/GPX4 pathway
Source: PLoS One. 2025 Jun 20;20(6):e0325674. doi: 10.1371/journal.pone.0325674 (PMC12180730; doi:10.1371/journal.pone.0325674)
Supplement: S1 Data — (PDF) [file pone.0325674.s001.pdf]

Figure1a Body weight (g)									
		Control	T2DM	PC	PMSL	PMSM	PMSH		
0	1	49.7	56.6	47.1	42	45.5	50.6		
	2	53.3	46.6	44.3	55.7	40.4	55		
	3	39.8	43.6	53.6	49.7	54.5	47.3		
	4	50.9	53.4	43	50	47.9	46.8		
	5	45	47.6	50.8	52.2	42	38.1		
	6	45.2	49.5	39.4	41.2	39.2	42.2		
	7	54.1	39.8	42.8	44.8	49.5	40.6		
	8	56.3	44.1	45.4	39.6	50.4	54.7		
	9	48	42.3	42	50.6	48	46.3		
	10	53.2	43.9	46.7	42	44.8	42		
2	1	48	45.7	40.7	51.6	44.1	41.1		
	2	53.5	52.6	42.1	49.8	46.8	38.4		
	3	45.4	40.3	48.2	43.3	48.4	41.7		
	4	44	42.3	44.3	46.6	44.9	49.8		
	5	51.7	45.7	45.9	39	53.7	52.1		
	6	44.3	50.1	43.6	46.1	44.4	37.7		
	7	44.6	32.2	49.3	43.8	40.2	53.9		
	8	47.7	44.3	45.4	53.2	44.5	48.7		
	9	45.5	34.2	49.8	40.2	41.8	33.5		
	10	51.8	49.6	47.4	35.9	38	51.5		
4	1	45.6	41.4	45	42.1	38.1	42.5		
	2	45.2	43	48	43.4	43.1	43.5		
	3	49.6	41.3	47.4	50.1	45.5	51.5		
	4	46.5	40.8	34.5	46.6	50.4	50.3		
	5	57.9	38.6	47.4	45.5	50.9	44		
	6	40.8	37.1	42.7	43	44.9	49.2		
	7	45.1	42.8	48.4	43.3	51.3	52.7		
	8	54.2	42	44.8	45.6	44.8	45.8		
	9	58.2	47.6	48.6	41.9	36.2	38.5		
	10	49.3	42.9	48.1	37.8	39.2	45.4		
6	1	54.9	37.7	35.7	35.5	41.1	46		
	2	38.5	37.2	42.9	41.3	37.5	48.8		
	3	44.4	36.6	41.2	37.8	45.3	45.7		
	4	51.2	36.6	49.4	42.7	51.7	36.9		
	5	51.6	35.3	50.8	39.2	44.8	41.1		
	6	42.9	35.7	41.5	41.4	39.7	43		
	7	52.9	38.7	57.6	43.9	48.2	48.2		
	8	52.6	37.9	45	45.5	40.5	50.7		
	9	44.7	35.2	44.5	43.8	42.5	53.1		
	10	49.2	38.9	39.9	47.4	41.1	41.2		
8	1	51.9	37.9	34.3	38.2	36.3	45.6		
	2	50.4	42.9	51.3	38.4	35.4	41.7		
	3	47.3	33.2	43.8	38.3	43.3	45.2		
	4	52.8	34.3	44.3	32.5	40	36.3		
	5	49.5	30.1	46.4	41.9	42.3	48.4		
	6	48.4	32.7	46.1	39.4	38.4	50.9		
	7	39.9	27.4	47.8	35.3	48.1	41.6		
	8	52.7	42.6	42.4	42.7	39.5	42.5		
	9	50.1	38.9	40.9	41.5	43.6	40.2		
	10	44.2	39.7	41.6	39	40.8	44.4		
Figure1b FBG (mmol/L)									
Weeks		Control	T2DM	PC	PMSL	PMSM	PMSH		
0	1	5	35.4	23.9	25.2	30.6	24.9		
	2	6.4	28.5	32.7	33.5	37.9	32.1		
	3	5.8	28.9	26.4	28.3	30.5	31.5		
	4	5.2	28	29.2	33.4	27.4	28.2		
	5	6.2	34.5	32.4	32.1	28.3	35.3		
	6	4.8	25.6	26.1	27.6	27.3	25.7		
	7	5.6	31.6	28.8	33.6	27.8	30		
	8	6.3	30.8	29.7	30.8	29.7	34.1		
	9	5.1	30.2	32.6	30.3	28	29.6		
	10	5.7	30.5	30.9	24.9	27	28.7		
2	1	5.4	24.6	27.5	27.1	26.7	26.2		
	2	5.5	30.4	24.2	28.4	30.2	26.8		
	3	5.7	30.4	27.2	26.4	30.7	29.8		
	4	6.4	30	29.9	31.5	25.1	27.3		
	5	5	28.1	23.9	29.2	26.5	30.6		
	6	5.3	29.4	24.7	32.6	30.5	28.7		
	7	5.9	31.5	29.4	32.6	27.4	27.9		
	8	5.6	32.6	26.4	32.7	32.2	26.6		
	9	5.6	38.1	30.4	32.8	22.8	28.9		
	10	5.3	34.4	24	26.1	25.9	28.2		
4	1	5.3	33.8	24.6	26.4	25.3	28.7		
	2	5.1	31.5	29.9	27.9	33.4	30		
	3	6.1	32.3	25.8	38.7	25.4	29		

	4	5.5	32.1	26.9	32.7	24.9	28.1			
	5	4	31.2	21.9	30.6	26	30.3			
	6	5.8	27.2	26.2	30.7	25.5	21.9			
	7	6.2	34.7	27.8	30.6	32.8	27.4			
	8	5.7	32.5	30.1	23.4	26.1	21.8			
	9	5.9	31.5	22.2	31.7	29	24.6			
	10	4.3	33.2	25.7	35.7	28.6	27.3			
6	1	5.7	28.4	22.5	30.3	25.3	25.6			
	2	5.8	29.8	24.1	26.8	25.1	19.9			
	3	4.8	28.4	18.2	36.5	20.4	23.6			
	4	4.6	37.4	22.3	29.5	26.6	22.5			
	5	6.1	29.9	20.4	32.4	21.7	23.7			
	6	4.8	28.1	25.6	31.8	25.8	24.5			
	7	5.6	34.5	25.5	25	28.6	24.5			
	8	5.2	33.6	23.1	27.4	26.2	24.1			
	9	5.3	36	22.7	25.7	21.3	25.7			
	10	4.8	28.5	20.9	30	27.7	19.7			
8	1	5.5	29.4	20.2	26.7	26.4	24.8			
	2	5	32.8	19.1	30.3	19.5	25			
	3	5.7	26.1	20.2	31.7	26	19.2			
	4	4.7	33	23.4	28.5	20.4	17.8			
	5	4.8	34.7	22	30.7	21.4	25			
	6	5.6	31.7	20.8	33.9	25.6	21			
	7	5.8	34.6	20.9	26.7	24.7	19.4			
	8	5.3	26.9	20.3	27.1	24.3	23.1			
	9	5	35	19.1	30.4	18.7	21.9			
	10	5.8	39.9	19.5	33.9	27.1	21.5			
**Figure1c HbAc (mg/mL)**										
	Control1	23.6		T2DM1	39.5		PC1	32.6		
	Control2	23.6		T2DM2	33.7		PC2	25.8		
	Control3	12.9		T2DM3	23.1		PC3	22.5		
	Control4	21.4		T2DM4	27.4		PC4	21.2		
	Control5	16.8		T2DM5	23.6		PC5	26.2		
	Control6	23.4		T2DM6	40.7		PC6	28.6		
	Control7	21.8		T2DM7	38.4		PC7	20.5		
	Control8	22.5		T2DM8	34.1		PC8	22.5		
	Control9	20.0		T2DM9	35.0		PC9	22.5		
	Control10	21.8		T2DM10	35.2		PC10	27.1		
	PMSL1	25.7		PMSM1	28.0		PMSH1	19.8		
	PMSL2	28.8		PMSM2	24.7		PMSH2	17.5		
	PMSL3	33.6		PMSM3	32.3		PMSH3	31.6		
	PMSL4	29.1		PMSM4	28.2		PMSH4	22.4		
	PMSL5	35.1		PMSM5	31.5		PMSH5	26.8		
	PMSL6	23.9		PMSM6	32.9		PMSH6	19.8		
	PMSL7	34.8		PMSM7	29.3		PMSH7	33.2		
	PMSL8	27.5		PMSM8	23.6		PMSH8	29.0		
	PMSL9	30.5		PMSM9	24.3		PMSH9	26.3		
	PMSL10	40.9		PMSM10	20.3		PMSH10	25.1		
**Figure1d e AUC of OGTT**										
	Control1	Control2	Control3	Control4	Control5	Control6	Control7	Control8	Control9	Control10
0	5.1	5.4	7.1	5.8	4.8	4.1	5.3	5.2	5.8	8.3
15	9.7	10.4	8.1	8.5	8.5	7.2	11.4	9.2	11.2	13.1
30	16.9	14.7	14.1	16.9	16.9	10.6	15.9	14.7	15.4	9.8
60	10.9	7.6	9	13	13	13.7	10	12.9	10.9	15.2
90	9.9	8.4	11.1	8.7	8.1	10.2	8.8	11.9	6.9	9.5
120	7.2	8	7.5	7.7	6.2	6.9	7.6	6.9	9.3	9.9
	T2DM1	T2DM2	T2DM3	T2DM4	T2DM5	T2DM6	T2DM7	T2DM8	T2DM9	T2DM10
0	38.4	34.9	27.5	28	26.4	32.9	26.3	38	36.6	38.7
15	39.7	27.3	31	35.9	37.3	31.2	28.9	37.8	26.4	39.9
30	38.7	33.9	36.8	45.9	38.7	34.4	21.2	36.7	24.2	36.8
60	29	38.9	27.4	30.8	33.1	30.5	38.8	35.4	34.2	29.6
90	34.7	31.4	33.4	30.9	39.5	32.7	29.6	26.3	32.1	35.2
120	31.8	27.3	34.7	26	33	37.9	34.4	27	32.5	28.8
	PC1	PC2	PC3	PC4	PC5	PC6	PC7	PC8	PC9	PC10
0	13.8	24.3	27.5	27.7	24.7	13.5	17.5	12.5	19.3	20.1
15	22.7	10.8	26.6	28.1	27	25.3	27.7	31.5	24.6	23.3
30	38.5	25.5	30.8	24.9	37.5	29.3	22	35.6	34.2	29.4
60	28.5	34.6	20.8	22	29.6	38.7	28.7	30.9	26.2	23.4
90	15.2	28.5	33.2	20.2	30.3	26	17.6	28.2	30	34.3
120	17.4	15.1	24.8	32.6	26.8	25.4	29.5	21.1	25.7	21.6
	2882	3038	3290	2945	3633	3462	2873	3457	3355	3217
	PMSL1	PMSL2	PMSL3	PMSL4	PMSL5	PMSL6	PMSL7	PMSL8	PMSL9	PMSL10
0	23.9	28.5	28.3	32.8	27.1	23.3	32.5	38.4	21.7	35.4
15	25.8	31	32.8	28.6	34.9	25.4	40.9	37.1	34.4	26.4
30	27.1	31	35	27.7	33.9	32.6	38.2	29.5	34.5	30.5
60	27.3	22.5	32.2	31.8	24.5	27.8	37.5	27.1	35.8	33.9
90	22	20.7	28.1	32.7	31.3	33.4	34.6	29.1	26.8	35.7

120	23.6	32	26.4	16.5	27.6	32.2	32.1	30.5	28.6	30.5
	3009	3152	3697	3481	3578	3608	4361	3652	3762	3893
	PMSM1	PMSM2	PMSM3	PMSM4	PMSM5	PMSM6	PMSM7	PMSM8	PMSM9	PMSM10
0	18	28.2	22.2	30.5	29.4	22.3	18.8	26.3	17.8	26.7
15	26.4	21.9	29	28.6	21.3	29.2	25.9	28	33	33.3
30	39.3	30.6	29.1	38.5	36.7	26.2	33.5	20.8	32	27.4
60	22	25.8	34.7	28.1	38.7	30.3	38.2	24.2	18.1	34.3
90	31.4	17.5	25	27.1	23.2	28.7	16.8	33.1	32.3	30.7
120	25.5	23.5	30.1	27.6	29.3	23.6	24.2	17.7	25	32.1
	PMSH1	PMSH2	PMSH3	PMSH4	PMSH5	PMSH6	PMSH7	PMSH8	PMSH9	PMSH10
0	21.6	16.5	23.8	24.3	21.4	26.4	22.4	22.6	14.4	23.3
15	33.5	29.8	35.6	21.4	20.2	18.3	25.1	24.4	19	30.4
30	39.1	32.6	23.4	24	28.9	30	31.2	27.7	26	37.9
60	26.3	32.9	22.5	30	31.2	26.4	36.5	25.5	26.9	34.4
90	26.3	26.7	22.1	26.2	23.4	21.4	31.7	34.9	34.5	16.1
120	27.8	20.3	22.9	17.6	29.3	27.2	20.8	31.7	16.9	30.6
AUC（mM·h·L-1）										
	Control		T2DM	PC		PMSL		PMSM		PMSH
	1296		4142	2882		3009		3400		3539
	1127		3953	3038		3152		2880		3397
	1208		3844	3290		3697		3499		2921
	1318		4022	2945		3481		3594		2993
	1270		4301	3633		3578		3662		3191
	1198		3953	3462		3608		3319		2990
	1247		3676	2873		4361		3296		3605
	1355		3934	3457		3652		3070		3446
	1232		3692	3355		3762		3236		3074
	1369		4093	3217		3893		3748		3458
**Figure1f HOMA-IR**										
Blank	0.054	Control1	1.994		T2DM1	1.876			PC1	1.891
0	2.134	Control2	1.953		T2DM2	1.808			PC2	1.95
0.5	1.912	Control3	1.978		T2DM3	1.874			PC3	1.841
1	1.667	Control4	2.052		T2DM4	1.836			PC4	1.862
2.5	1.438	Control5	1.994		T2DM5	1.874			PC5	1.918
5	1.004	Control6	1.927		T2DM6	1.783			PC6	1.933
10	0.475	Control7	2.022		T2DM7	1.878			PC7	1.911
		Control8	1.908		T2DM8	1.871			PC8	1.984
		Control9	1.951		T2DM9	1.83			PC9	1.958
		Control10	2.013		T2DM10	1.802			PC10	1.959
		PMSL1	1.939		PMSM1	1.957			PMSH1	1.895
		PMSL2	1.778		PMSM2	1.868			PMSH2	1.914
		PMSL3	1.812		PMSM3	1.919			PMSH3	1.899
		PMSL4	1.902		PMSM4	1.911			PMSH4	1.874
		PMSL5	1.948		PMSM5	1.892			PMSH5	1.876
		PMSL6	1.89		PMSM6	1.798			PMSH6	1.901
		PMSL7	1.824		PMSM7	1.919			PMSH7	1.812
		PMSL8	1.926		PMSM8	1.887			PMSH8	1.938
		PMSL9	1.883		PMSM9	1.895			PMSH9	1.897
		PMSL10	1.831		PMSM10	1.845			PMSH10	1.998
**Figure2 d TUNEL**										
			IntDen	Area	Mean					
		Control1	608774	316694	1.922278288	1.608556476	0.013659171			
		Control2	587465	326547	1.799021274	1.505415392	0.012783341			
		Control3	501257	365417	1.371739684	1.147867489	0.009747198			
		T2DM1	54518425	316203	172.4159005	144.2770879	1.22513909			
		T2DM2	45681236	324517	140.7668504	117.7932614	1.000249806			
		T2DM3	39836268	365429	109.0123334	91.22118067	0.774611104			
		PC1	16087971	372171	43.22736323	36.17252274	0.307161534			
		PC2	17123684	365946	46.79292573	39.15617432	0.332497422			
		PC3	11754628	374162	31.41587868	26.28870931	0.223232433			
		PMSL1	46487938	393566	118.119802	98.84228196	0.839326224			
		PMSL2	35832176	413269	86.70424348	72.55384055	0.616096066			
		PMSL3	41736587	397412	105.0209531	87.88120601	0.746249473			
		PMSM1	41666540	453936	91.78945931	76.80913329	0.652230185			
		PMSM2	41362687	475632	86.96363365	72.77089743	0.61793922			
		PMSM3	32523695	471236	69.01784881	57.75392064	0.4904215			
		PMSH1	22293365	397287	56.11400574	46.95602501	0.398730406			
		PMSH2	19756327	357846	55.20902008	46.19873583	0.392299831			
		PMSH3	16742682	412736	40.56511184	33.94475908	0.288244321			
**Figure2 e ROS**										
			Control1	3929		T2DM1	8044		PC1	5669
			Control2	3979		T2DM2	7618		PC2	5512
			Control3	3644		T2DM3	8238		PC3	5304
			Control4	4711		T2DM4	7793		PC4	6479
			Control5	4453		T2DM5	7088		PC5	5909
			Control6	3622		T2DM6	8954		PC6	6937
			Control7	4084		T2DM7	6901		PC7	6297

			Control8	4105		T2DM8	8716		PC8	7023
			Control9	3753		T2DM9	8634		PC9	5270
			Control10	4286		T2DM10	6988		PC10	5656
			PMSL1	7537		PMSM1	5653		PMSH1	6560
			PMSL2	6418		PMSM2	5653		PMSH2	6014
			PMSL3	6830		PMSM3	5605		PMSH3	7156
			PMSL4	7311		PMSM4	6356		PMSH4	6202
			PMSL5	6713		PMSM5	6703		PMSH5	5767
			PMSL6	7121		PMSM6	6410		PMSH6	6239
			PMSL7	6918		PMSM7	6117		PMSH7	5540
			PMSL8	7404		PMSM8	5956		PMSH8	6356
			PMSL9	7005		PMSM9	6118		PMSH9	5428
			PMSL10	7225		PMSM10	7120		PMSH10	5066
**Figure2 f MDA**										
Blank	standard		Control1	0.357		T2DM1	0.936		PC1	0.702
0.041	0.106		Control2	0.288		T2DM2	0.861		PC2	0.636
0.043	0.103		Control3	0.333		T2DM3	0.843		PC3	0.708
0.046	0.104		Control4	0.321		T2DM4	0.9		PC4	0.702
0.046	0.102		Control5	0.345		T2DM5	0.762		PC5	0.629
			Control6	0.378		T2DM6	0.798		PC6	0.633
			Control7	0.39		T2DM7	0.756		PC7	0.735
			Control8	0.345		T2DM8	0.81		PC8	0.771
			Control9	0.315		T2DM9	0.744		PC9	0.663
			Control10	0.345		T2DM10	0.822		PC10	0.696
			PMSL1	0.864		PMSM1	0.75		PMSH1	0.606
			PMSL2	0.783		PMSM2	0.717		PMSH2	0.723
			PMSL3	0.795		PMSM3	0.69		PMSH3	0.708
			PMSL4	0.669		PMSM4	0.795		PMSH4	0.765
			PMSL5	0.768		PMSM5	0.807		PMSH5	0.75
			PMSL6	0.828		PMSM6	0.723		PMSH6	0.766
			PMSL7	0.765		PMSM7	0.765		PMSH7	0.739
			PMSL8	0.813		PMSM8	0.624		PMSH8	0.684
			PMSL9	0.777		PMSM9	0.795		PMSH9	0.654
			PMSL10	0.837		PMSM10	0.717		PMSH10	0.738
**Figure2 g 4-HNE**										
Blank	0.044		Control1	2.121		T2DM1	2.603		PC1	2.018
2000	0.115		Control2	2.082		T2DM2	2.715		PC2	2.072
1000	0.393		Control3	2.121		T2DM3	2.609		PC3	2.546
500	0.692		Control4	2.17		T2DM4	2.513		PC4	2.171
250	0.971		Control5	1.892		T2DM5	2.449		PC5	2.519
125	1.129		Control6	2.219		T2DM6	2.712		PC6	1.976
62.5	1.805		Control7	2.005		T2DM7	2.406		PC7	2.358
			Control8	1.819		T2DM8	2.647		PC8	2.25
			Control9	2.105		T2DM9	2.218		PC9	2.218
			Control10	2.139		T2DM10	2.277		PC10	2.202
			PMSL1	2.219		PMSM1	2.297		PMSH1	2.321
			PMSL2	2.651		PMSM2	2.094		PMSH2	2.258
			PMSL3	2.369		PMSM3	2.575		PMSH3	2.413
			PMSL4	1.982		PMSM4	2.316		PMSH4	2.176
			PMSL5	2.382		PMSM5	2.442		PMSH5	2.137
			PMSL6	2.2		PMSM6	2.387		PMSH6	2.364
			PMSL7	2.375		PMSM7	2.413		PMSH7	2.443
			PMSL8	2.99		PMSM8	2.39		PMSH8	2.504
			PMSL9	2.711		PMSM9	2.214		PMSH9	2.227
			PMSL10	2.598		PMSM10	2.209		PMSH10	2.233
**Figure2 h iron**										
Blank	standard		Control1	0.111		T2DM1	0.335		PC1	0.217
0.041	0.106		Control2	0.098		T2DM2	0.297		PC2	0.193
0.043	0.103		Control3	0.123		T2DM3	0.254		PC3	0.191
0.046	0.104		Control4	0.115		T2DM4	0.309		PC4	0.214
0.046	0.102		Control5	0.133		T2DM5	0.284		PC5	0.243
			Control6	0.132		T2DM6	0.248		PC6	0.225
			Control7	0.119		T2DM7	0.265		PC7	0.217
			Control8	0.101		T2DM8	0.268		PC8	0.196
			Control9	0.127		T2DM9	0.247		PC9	0.213
			Control10	0.105		T2DM10	0.268		PC10	0.211
			PMSL1	0.301		PMSM1	0.283		PMSH1	0.221
			PMSL2	0.225		PMSM2	0.213		PMSH2	0.267
			PMSL3	0.277		PMSM3	0.201		PMSH3	0.248
			PMSL4	0.252		PMSM4	0.232		PMSH4	0.257
			PMSL5	0.261		PMSM5	0.236		PMSH5	0.241
			PMSL6	0.295		PMSM6	0.271		PMSH6	0.215
			PMSL7	0.255		PMSM7	0.232		PMSH7	0.256
			PMSL8	0.246		PMSM8	0.246		PMSH8	0.224
			PMSL9	0.29		PMSM9	0.229		PMSH9	0.229
			PMSL10	0.26		PMSM10	0.263		PMSH10	0.222
**Figure2 i—l**										

		1			2			3		
	Actb	ΔCT								
	Control	19.384	19.360	19.372	19.386	19.378	19.330	19.388	19.366	19.382
	T2DM	19.378	19.377	19.388	19.412	19.413	19.410	19.337	19.348	19.367
	PC	19.377	19.369	19.400	19.362	19.391	19.350	19.363	19.371	19.381
	PMSL	19.344	19.352	19.382	19.367	19.401	19.355	19.371	19.316	19.363
	PMSM	19.368	19.367	19.395	19.354	19.367	19.365	19.368	19.362	19.364
	PMSH	19.369	19.380	19.388	19.410	19.349	19.385	19.356	19.379	19.359
	Ascl4	ΔCT								
	Control	23.254	23.208	23.217	23.358	23.311	23.322	23.27	23.23	23.173
	T2DM	22.343	22.272	22.302	22.321	22.315	22.291	22.348	22.386	22.305
	PC	23.091	23.036	23.039	23.011	23.033	23.013	23.142	23.161	23.162
	PMSL	22.549	22.534	22.551	22.457	22.452	22.418	22.574	22.534	22.533
	PMSM	22.734	22.753	22.773	22.76	22.742	22.77	22.892	22.839	22.847
	PMSH	22.905	22.905	22.889	22.808	22.819	22.853	22.911	22.989	22.988
	Ftl1	ΔCT								
	Control	22.331	22.338	22.346	22.303	22.306	22.315	22.325	22.359	22.322
	T2DM	24.19	24.179	24.233	24.191	24.183	24.219	24.277	24.235	24.266
	PC	23.25	22.245	22.243	22.242	22.204	23.265	22.922	23.122	21.909
	PMSL	23.998	24.036	24.032	24.013	24.048	24.041	23.831	24	23.913
	PMSM	23.629	23.657	23.636	23.691	23.604	23.6	23.786	23.733	23.755
	PMSH	23.208	23.181	23.2	23.208	23.212	23.175	23.319	23.322	23.298
	Trf	ΔCT								
	Control	23.329	23.363	23.331	23.392	23.464	23.442	23.241	23.249	23.224
	T2DM	22.313	22.311	22.33	22.297	22.286	22.323	22.316	22.312	22.313
	PC	23.047	22.969	22.999	23.046	23.052	23.013	23.144	23.158	23.105
	PMSL	22.533	22.545	22.578	22.472	22.423	22.433	22.41	22.414	22.455
	PMSM	22.65	22.638	22.667	22.699	22.651	22.66	22.532	22.541	22.561
	PMSH	22.771	22.805	22.762	22.911	22.913	22.886	22.89	22.899	22.927
	Steap3	ΔCT								
	Control	23.212	23.241	23.203	23.325	23.321	23.338	23.432	23.453	23.439
	T2DM	22.216	22.178	22.207	22.241	22.239	22.186	22.313	22.301	22.287
	PC	22.922	22.957	22.918	23.224	23.627	23.018	23.03	23.31	23.019
	PMSL	22.465	21.981	22.444	22.379	22.339	22.367	22.451	22.152	22.441
	PMSM	22.038	22.833	22.056	22.66	22.169	22.081	22.656	21.66	22.347
	PMSH	22.884	22.916	22.903	22.791	21.992	22.982	22.888	22.072	22.918
**Figure2 n—q**										
		ACSL4	β-actin		ACSL4	β-actin		ACSL4	β-actin	
	Control	15168	84180		17346	79855		18599	80601	
	T2DM	110039	90032		108135	88191		142921	83519	
	PC	63158	86429		62709	82792		69112	78596	
	PMSL	107052	85853		109787	89744		121323	78014	
	PMSM	86329	81524		76391	80491		96026	76425	
	PMSH	52769	66046		53167	64526		62639	63570	
		FTL	β-actin		FTL	β-actin		FTL	β-actin	
	Control	162557	84180		180043	79855		224486	80601	
	T2DM	79964	90032		97878	88191		96017	83519	
	PC	168320	86429		184738	82792		217289	78596	
	PMSL	119062	85853		94571	89744		129112	78014	
	PMSM	136939	81524		153009	80491		173913	76425	
	PMSH	131785	66046		132597	64526		157833	63570	
		Transferrin	β-actin		Transferrin	β-actin		Transferrin	β-actin	
	Control	24942	84180		24913	79855		21056	80601	
	T2DM	89151	90032		90775	88191		73742	83519	
	PC	30458	86429		34061	82792		25550	78596	
	PMSL	84345	85853		80490	89744		73553	78014	
	PMSM	64829	81524		70323	80491		61898	76425	
	PMSH	44886	66046		50030	64526		38921	63570	
		steap3	β-actin		steap3	β-actin		steap3	β-actin	
	Control	28645	84180		29350	79855		23796	80601	
	T2DM	91326	90032		111775	88191		104533	83519	
	PC	42848	86429		50408	82792		40122	78596	
	PMSL	67992	85853		67270	89744		53470	78014	
	PMSM	59331	81524		62262	80491		51897	76425	
	PMSH	45872	66046		48051	64526		38158	63570	
**Figure3 a-c**										
T-GSH	A1	A2	GSSG	A1	A2	GSH			GSH/GSSG	
Control1	0.405	1.651	Control1	0.15	0.318	Control1	10.8347692		Control1	7.00942623
Control2	0.322	1.4	Control2	0.195	0.308	Control2	9.482184133		Control2	9.588539098
Control3	0.327	1.637	Control3	0.191	0.326	Control3	10.44232278		Control3	9.787613843
Control4	0.367	1.226	Control4	0.168	0.336	Control4	6.572651027		Control4	4.211153396
Control5	0.369	1.616	Control5	0.173	0.346	Control5	10.89380714		Control5	6.756059888
Control6	0.422	1.611	Control6	0.183	0.308	Control6	11.55781427		Control6	9.554740984
Control7	0.4	1.726	Control7	0.191	0.263	Control7	13.50269738		Control7	20.37172131

Control8	0.554	1.468	Control8	0.185	0.308	Control8	9.388644723		Control8	7.026709316
Control9	0.426	1.077	Control9	0.179	0.368	Control9	4.205362202		Control9	2.184153005
Control10	0.39	1.558	Control10	0.203	0.324	Control10	11.16226621		Control10	9.725890801
T2DM1	0.365	1.465	T2DM1	0.194	0.436	T2DM1	8.182681866		T2DM1	3.521609538
T2DM2	0.451	1.22	T2DM2	0.198	0.374	T2DM2	5.461418451		T2DM2	3.307647168
T2DM3	0.442	0.918	T2DM3	0.182	0.419	T2DM3	0.982594463		T2DM3	0.439759286
T2DM4	0.38	1.149	T2DM4	0.173	0.371	T2DM4	4.765299591		T2DM4	2.717908594
T2DM5	0.461	1.332	T2DM5	0.197	0.4	T2DM5	6.150784702		T2DM5	3.212073003
T2DM6	0.424	0.732	T2DM6	0.17	0.352	T2DM6	0.094498576		T2DM6	0.055737705
T2DM7	0.421	0.827	T2DM7	0.161	0.366	T2DM7	0.818396467		T2DM7	0.405805678
T2DM8	0.451	1.116	T2DM8	0.149	0.448	T2DM8	1.790587756		T2DM8	0.70171062
T2DM9	0.352	0.993	T2DM9	0.179	0.43	T2DM9	2.463763749		T2DM9	1.102220626
T2DM10	0.373	1.156	T2DM10	0.192	0.426	T2DM10	3.898926969		T2DM10	2.064754098
PC1	0.414	1.389	PC1	0.165	0.348	PC1	7.329853928		PC1	4.472050524
PC2	0.373	1.285	PC2	0.172	0.347	PC2	6.241649188		PC2	4.330604215
PC3	0.443	1.581	PC3	0.154	0.33	PC3	7.96018341		PC3	5.854489568
PC4	0.38	1.596	PC4	0.168	0.324	PC4	10.31736158		PC4	7.468852459
PC5	0.43	1.124	PC5	0.179	0.324	PC5	5.222539901		PC5	3.814064443
PC6	0.454	1.614	PC6	0.182	0.361	PC6	10.09227861		PC6	5.872149464
PC7	0.376	1.532	PC7	0.175	0.347	PC7	9.245788811		PC7	6.164277545
PC8	0.372	1.689	PC8	0.172	0.319	PC8	11.97631339		PC8	8.883205085
PC9	0.396	1.39	PC9	0.196	0.287	PC9	10.70023469		PC9	11.26885246
PC10	0.408	1.528	PC10	0.174	0.317	PC10	9.341798314		PC10	7.514157973
PMSL1	0.402	1.207	PMSL1	0.167	0.385	PMSL1	4.993743588		PMSL1	2.485674538
PMSL2	0.447	1.253	PMSL2	0.169	0.437	PMSL2	4.500574285		PMSL2	1.653327624
PMSL3	0.432	0.854	PMSL3	0.172	0.382	PMSL3	0.863324517		PMSL3	0.441077283
PMSL4	0.415	1.37	PMSL4	0.17	0.431	PMSL4	5.796547341		PMSL4	2.4447899
PMSL5	0.383	0.906	PMSL5	0.179	0.421	PMSL5	1.243254913		PMSL5	0.625274353
PMSL6	0.435	1.288	PMSL6	0.182	0.339	PMSL6	7.040180405		PMSL6	4.599906025
PMSL7	0.375	1.143	PMSL7	0.2	0.326	PMSL7	6.819695407		PMSL7	5.404215457
PMSL8	0.402	1.595	PMSL8	0.187	0.441	PMSL8	8.390531431		PMSL8	3.705518265
PMSL9	0.472	1.305	PMSL9	0.174	0.427	PMSL9	4.186525578		PMSL9	1.999565865
PMSL10	0.447	1.708	PMSL10	0.165	0.391	PMSL10	8.624742702		PMSL10	4.777897867
PMSM1	0.476	1.508	PMSM1	0.152	0.418	PMSM1	6.367076533		PMSM1	2.712880562
PMSM2	0.412	1.028	PMSM2	0.198	0.346	PMSM2	4.490557912		PMSM2	3.056003545
PMSM3	0.435	1.544	PMSM3	0.168	0.395	PMSM3	7.405673906		PMSM3	3.934635661
PMSM4	0.415	0.935	PMSM4	0.195	0.425	PMSM4	1.665041769		PMSM4	0.74640057
PMSM5	0.387	1.325	PMSM5	0.182	0.382	PMSM5	6.724328419		PMSM5	3.697196721
PMSM6	0.418	1.535	PMSM6	0.159	0.419	PMSM6	7.264728398		PMSM6	3.218770492
PMSM7	0.432	1.367	PMSM7	0.163	0.34	PMSM7	6.52948851		PMSM7	4.416921367
PMSM8	0.369	1.21	PMSM8	0.168	0.309	PMSM8	8.064853488		PMSM8	5.245448204
PMSM9	0.435	1.389	PMSM9	0.182	0.34	PMSM9	7.395154026		PMSM9	5.334654493
PMSM10	0.454	1.569	PMSM10	0.168	0.387	PMSM10	9.551129315		PMSM10	4.184706939
PMSH1	0.433	1.489	PMSH1	0.166	0.367	PMSH1	8.620438478		PMSH1	4.381991681
PMSH2	0.436	1.66	PMSH2	0.181	0.329	PMSH2	9.687223339		PMSH2	8.046344705
PMSH3	0.323	1.034	PMSH3	0.178	0.341	PMSH3	4.910428801		PMSH3	3.298712662
PMSH4	0.397	1.434	PMSH4	0.17	0.382	PMSH4	8.404001198		PMSH4	3.941981132
PMSH5	0.369	1.632	PMSH5	0.172	0.363	PMSH5	10.49486016		PMSH5	6.032640975
PMSH6	0.403	1.495	PMSH6	0.173	0.415	PMSH6	7.545940104		PMSH6	3.481452378
PMSH7	0.406	1.248	PMSH7	0.163	0.368	PMSH7	4.894810335		PMSH7	2.989380248
PMSH8	0.397	1.524	PMSH8	0.157	0.36	PMSH8	7.617714274		PMSH8	4.74397965
PMSH9	0.419	1.498	PMSH9	0.168	0.319	PMSH9	8.72120362		PMSH9	6.680262729
PMSH10	0.34	1.291	PMSH10	0.158	0.318	PMSH10	6.749620299		PMSH10	5.220194672
						**Figure3 d-f**				
		**1**			**2**			**3**		
	**Actb**	ΔCT								
	Control	19.327	19.358	19.340	19.313	19.345	19.357	19.342	19.328	19.399
	T2DM	19.423	19.351	19.376	19.367	19.349	19.345	19.366	19.335	19.374
	PC	19.443	19.412	19.431	19.384	19.362	19.360	19.375	19.388	19.360
	PMSL	19.429	19.395	19.404	19.371	19.361	19.342	19.335	19.354	19.354
	PMSM	19.433	19.418	19.428	19.376	19.387	19.355	19.368	19.369	19.355
	PMSH	19.351	19.376	19.354	19.393	19.337	19.362	19.381	19.321	19.366
	**Slc3a2**	ΔCT								
	Control	21.964	21.524	20.529	21.345	21.33	21.32	21.315	21.332	22.055
	T2DM	23.121	23.125	23.184	23.223	23.23	23.2	23.228	23.13	23.232
	PC	21.962	21.261	21.981	21.3334	22.194	21.169	22.121	21.116	22.131
	PMSL	22.999	22.965	22.949	23.111	23.275	23.105	22.889	22.397	22.906
	PMSM	22.758	22.807	22.785	22.972	22.762	22.968	22.662	22.175	22.634
	PMSH	22.511	22.145	22.537	22.452	22.417	22.404	22.554	21.312	22.508
	**Slc7a11**									
	Control	21.259	21.234	21.241	21.213	21.207	21.246	21.636	21.629	21.629
	T2DM	23.316	23.293	23.306	23.432	23.449	23.411	23.3	23.312	23.321
	PC	21.912	21.959	21.956	22.198	22.182	22.176	22.148	22.148	22.117
	PMSL	22.998	22.975	23.012	22.956	22.995	22.976	22.914	22.871	22.92
	PMSM	22.67	22.696	22.639	22.754	22.781	22.766	22.723	22.762	22.734
	PMSH	22.444	22.425	22.437	22.278	22.275	22.223	22.471	22.446	22.427
	**Gpx4**									

	Control	21.022	21.126	21.438	21.23	21.259	21.233	21.304	21.921	21.628
	T2DM	23.236	23.207	23.294	23.388	23.405	23.436	23.319	23.332	23.278
	PC	21.96	21.97	21.567	20.162	22.21	22.204	21.8141	22.27	21.052
	PMSL	22.976	22.001	23.008	22.236	22.096	23.004	22.185	23.389	22.221
	PMSM	22.688	22.66	22.68	22.141	22.737	22.75	22.854	21.122	22.852
	PMSH	22.442	22.034	22.431	22.556	22.066	22.538	22.334	21.644	22.007
**Figure3 h-j**										
		SLC7A11	β-actin		SLC7A11	β-actin		SLC7A11	β-actin	
	Control	262393	67079		220495	67009		207871	67260	
	T2DM	44146	66156		35225	66052		28878	67190	
	PC	226344	71437		199228	70530		180040	74195	
	PMSL	87976	68365		66967	68423		66246	70809	
	PMSM	162713	65404		157034	65672		109140	70954	
	PMSH	266408	58032		258816	58278		201705	58051	
**Figure4 a-b**										
	PMS	0	10	20	40	80	160			
	1	1.914	2.156	2.119	1.941	1.949	1.75			
	2	2.208	2.074	2.015	1.805	1.909	2.004			
	3	1.995	1.96	2.046	1.981	2.039	1.712			
	4	1.879	1.901	2.003	2.093	1.879	1.844			
	5	2.016	2.125	1.803	1.979	1.914	1.962			
	6	2.382	2.262	2.032	1.886	1.848	1.878			
	HGPA+PMS	Control	HGPA	PMS-10	PMS-20	PMS-40				
	1	1.955	1.459	1.995	1.963	1.804				
	2	2.32	1.619	1.874	1.704	2.124				
	3	2.045	1.527	1.801	2.063	1.905				
	4	1.885	1.369	1.507	1.802	2.067				
	5	2.061	1.502	1.617	2.001	1.628				
	6	2.291	1.438	1.584	1.501	1.955				
**Figure4 c -f**										
ROS	OD		MDA	OD		4-HNE	OD		iron	OD
Control1	3076		Control1	0.234		Control1	1.754		Control1	0.643
Control2	3288		Control2	0.315		Control2	1.792		Control2	0.64
Control3	2715		Control3	0.224		Control3	1.754		Control3	0.659
Control4	3535		Control4	0.284		Control4	1.715		Control4	0.563
Control5	2984		Control5	0.247		Control5	1.673		Control5	0.737
Control6	3113		Control6	0.258		Control6	2.06		Control6	0.704
HGPA1	7646		HGPA1	0.883		HGPA1	2.417		HGPA1	1.054
HGPA2	9021		HGPA2	0.845		HGPA2	2.359		HGPA2	1.454
HGPA3	8505		HGPA3	0.962		HGPA3	2.123		HGPA3	1.67
HGPA4	7871		HGPA4	0.729		HGPA4	2.309		HGPA4	1.29
HGPA5	7010		HGPA5	0.709		HGPA5	2.251		HGPA5	1.502
HGPA6	8338		HGPA6	0.688		HGPA6	2.259		HGPA6	1.642
PMS-40-1	5964		PMS-40-1	0.538		PMS-40-1	2.257		PMS-40-1	0.91
PMS-40-2	5408		PMS-40-2	0.639		PMS-40-2	1.978		PMS-40-2	1.379
PMS-40-3	7029		PMS-40-3	0.492		PMS-40-3	2.107		PMS-40-3	0.933
PMS-40-4	4018		PMS-40-4	0.534		PMS-40-4	1.999		PMS-40-4	1.596
PMS-40-5	6536		PMS-40-5	0.678		PMS-40-5	1.974		PMS-40-5	0.874
PMS-40-6	3649		PMS-40-6	0.705		PMS-40-6	1.912		PMS-40-6	0.831
**Figure4 g-j**										
		**1**			**2**			**3**		
	**Actb**									
	Control	18.452	18.461	18.412	18.477	18.423	18.449	18.437	18.441	18.452
	HGPA	18.430	18.455	18.432	18.458	18.455	18.490	18.478	18.432	18.490
	PMS-40	18.467	18.460	18.455	18.452	18.490	18.472	18.434	18.467	18.477
	**Ascl4**									
	Control	23.163	23.124	23.166	23.246	23.268	23.271	23.155	23.096	23.147
	HGPA	22.404	22.397	22.438	22.321	22.312	22.293	22.331	22.334	22.304
	PMS-40	22.79	22.826	22.829	22.921	22.923	22.933	22.823	22.793	22.811
	**Ftl1**									
	Control	22.819	22.874	22.81	22.933	22.941	23.463	22.786	22.806	22.83
	HGPA	23.977	23.932	23.979	23.946	23.901	23.905	23.948	23.991	23.919
	PMS-40	23.126	23.14	23.104	23.113	23.104	23.132	23.213	23.211	23.341
	**Trf**									
	Control	23.165	23.14	23.15	23.455	23.417	23.447	23.352	23.316	23.363
	HGPA	22.296	22.32	22.332	22.352	22.306	22.353	22.257	22.211	22.229
	PMS-40	22.818	22.824	22.836	22.801	22.838	22.829	22.715	22.689	22.628
	**Steap3**									
	Control	23.001	22.956	23.038	23.234	23.233	23.279	23.154	22.871	23.157
	HGPA	22.333	22.329	22.312	22.323	22.339	22.343	22.224	22.213	22.237
	PMS-40	22.8	22.805	22.797	22.716	22.743	22.718	22.838	23.962	22.821
**Figure4 l-o**										
		ACSL4	β-actin		ACSL4	β-actin			ACSL4	β-actin
	Control	68972	114503		74465	117161			74465	117161

	HGPA	242880	113687		263038	112330			263038	112330
	PMS-40	100598	105979		127967	100980			127967	100980
		FTL	β-actin		FTL	β-actin			FTL	β-actin
	Control	173033	114503		174981	117161			181374	143921
	HGPA	52310	113687		78636	112330			81744	150961
	PMS-40	143339	105979		149148	100980			152634	108783
		Transferrin	β-actin		Transferrin	β-actin			Transferrin	β-actin
	Control	78122	114503		64909	117161			63899	143921
	HGPA	223277	113687		195139	112330			199096	150961
	PMS-40	85843	105979		107052	100980			95486	108783
		steap3	β-actin		steap3	β-actin			steap3	β-actin
	Control	76237	114503		78067	117161			72569	143921
	HGPA	142273	113687		142456	112330			168765	150961
	PMS-40	81906	105979		91847	100980			86332	108783
**Figure5 a-c**										
T-GSH	A1	A2	GSSG	A1	A2	GSH			GSH/GSSG	
	0.052	0.244		0.149	0.368	Control1	10.06877406		Control1	7.761413005
	0.051	0.229		0.154	0.383	Control2	8.424809181		Control2	7.188281272
	0.052	0.237		0.155	0.372	Control3	10.10467484		Control3	7.350473613
	0.052	0.236		0.148	0.373	Control4	7.430003003		Control4	5.33959711
Control1	0.383	1.769	Control1	0.163	0.334	Control5	8.90028192		Control5	4.653333864
Control2	0.354	1.445	Control2	0.198	0.341	Control6	10.34746601		Control6	12.5214427
Control3	0.341	1.723	Control3	0.183	0.361	HGPA1	2.116044723		HGPA1	1.07311838
Control4	0.345	1.314	Control4	0.168	0.327	HGPA2	3.236962717		HGPA2	2.160413335
Control5	0.362	1.489	Control5	0.172	0.376	HGPA3	-0.637507117		HGPA3	-0.326656752
Control6	0.444	1.698	Control6	0.202	0.306	HGPA4	2.951783948		HGPA4	1.160356368
HGPA1	0.372	1.038	HGPA1	0.167	0.428	HGPA5	0.159084137		HGPA5	0.06994249
HGPA2	0.498	1.22	HGPA2	0.187	0.396	HGPA6	-0.80461046		HGPA6	-0.379302886
HGPA3	0.416	0.837	HGPA3	0.184	0.487	PMS-40-1	5.534510797		PMS-40-1	3.605017738
HGPA4	0.363	1.145	HGPA4	0.17	0.468	PMS-40-2	4.09999908		PMS-40-2	3.024963837
HGPA5	0.454	0.894	HGPA5	0.182	0.438	PMS-40-3	7.434076186		PMS-40-3	5.299579011
HGPA6	0.397	0.759	HGPA6	0.173	0.442	PMS-40-4	4.632783066		PMS-40-4	2.486640215
PMS-40-1	0.422	1.283	PMS-40-1	0.173	0.358	PMS-40-5	3.379420713		PMS-40-5	1.940028463
PMS-40-2	0.351	1.198	PMS-40-2	0.173	0.376	PMS-40-6	6.240471637		PMS-40-6	3.735473412
PMS-40-3	0.42	1.511	PMS-40-3	0.142	0.322					
PMS-40-4	0.363	1.462	PMS-40-4	0.161	0.456					
PMS-40-5	0.448	1.207	PMS-40-5	0.165	0.397					
PMS-40-6	0.465	1.427	PMS-40-6	0.191	0.393					
**Figure5 d-f**										
	**1**			**2**			**3**			
**Actb**	ΔCT									
Control	18.452	18.464	18.434	18.441	18.465	18.448	18.458	18.428	18.477	18.448
HGPA	18.458	18.482	18.457	18.448	18.462	18.432	18.517	18.468	18.445	18.432
PMS-40	18.444	18.424	18.445	18.441	18.423	18.454	18.437	18.457	18.468	18.454
**Slc3a2**										
Control	22.848	22.841	22.824	22.816	22.847	22.843	23.187	23.162	23.112	22.843
HGPA	23.798	23.801	23.802	23.874	23.884	23.916	23.927	23.92	23.928	23.916
PMS-40	23.125	23.138	23.15	23.135	23.124	23.098	23.335	23.355	23.314	23.098
**Slc7a11**	ΔCT									
Control	22.824	22.85	22.806	22.811	22.83	22.843	22.946	22.929	22.939	22.843
HGPA	23.889	23.92	23.893	23.918	23.993	23.983	23.933	23.927	23.872	23.983
PMS-40	23.319	23.343	23.391	23.151	23.134	23.1	23.434	23.451	23.4137	23.1
**Gpx4**	ΔCT									
Control	22.955	22.928	22.952	22.84	22.836	22.852	22.821	22.828	22.85	22.852
HGPA	23.927	23.911	23.958	23.883	23.871	23.895	23.825	23.804	23.829	23.895
PMS-40	23.107	23.097	23.131	23.137	23.105	23.129	23.241	23.246	23.232	23.129
**Figure5 h-j**										
		SLC7A11	β-actin		SLC7A11	β-actin		SLC7A11	β-actin	
	Control	75261	109565		67812	110990		71087	109138	
	HGPA	48570	110475		41537	115138		46195	107209	
	PMS-40	65472	111962		61101	111899		60805	111236	
		SLC3A2	β-actin		SLC3A2	β-actin		SLC3A2	β-actin	
	Control	42452	109565		41791	110990		44924	109138	
	HGPA	20758	110475		21260	115138		15297	107209	
	PMS-40	32099	111962		31691	111899		34730	111236	
		GPX4	β-actin		GPX4	β-actin		GPX4	β-actin	
	Control	116457	109565		112227	110990		104289	109138	
	HGPA	33568	110475		23959	115138		17714	107209	
	PMS-40	97932	111962		93007	111899		84225	111236	
**Figure6 a**										

		Control		HGPA		HGPA+PMS-40		HGPA+Fer-1		HGPA+PMS-40+RSL-3
	1	1.945		1.484		1.68		1.661		1.252
	2	2.463		1.672		1.996		1.757		1.452
	3	2.054		1.563		1.797		2.082		1.647
	4	1.987		1.322		2.109		2.033		1.417
	5	1.943		1.389		1.58		1.848		1.385
	6	2.001		1.528		2.063		1.903		1.523
**Figure6 b-e**										
ROS	OD		MDA	OD		4-HNE	OD		iron	OD
Control1	3139		Control1	0.215		Control1	1.619		Control1	0.884
Control2	3322		Control2	0.325		Control2	1.816		Control2	0.994
Control3	2525		Control3	0.224		Control3	1.745		Control3	1.043
Control4	3607		Control4	0.258		Control4	1.89		Control4	0.873
Control5	2868		Control5	0.271		Control5	1.564		Control5	1.166
Control6	3427		Control6	0.241		Control6	1.939		Control6	1.179
HGPA1	8962		HGPA1	0.886		HGPA1	2.202		HGPA1	1.904
HGPA2	8178		HGPA2	0.831		HGPA2	2.204		HGPA2	2.239
HGPA3	8505		HGPA3	0.922		HGPA3	2.178		HGPA3	2.644
HGPA4	6905		HGPA4	0.699		HGPA4	2.249		HGPA4	2.094
HGPA5	7538		HGPA5	0.66		HGPA5	2.432		HGPA5	2.337
HGPA6	8095		HGPA6	0.749		HGPA6	2.467		HGPA6	2.617
HGPA+PMS-40-1	5522		HGPA+PMS-40-1	0.564		HGPA+PMS-40-1	2.104		HGPA+PMS-40-1	1.272
HGPA+PMS-40-2	5355		HGPA+PMS-40-2	0.643		HGPA+PMS-40-2	2.081		HGPA+PMS-40-2	1.307
HGPA+PMS-40-3	4172		HGPA+PMS-40-3	0.455		HGPA+PMS-40-3	2.089		HGPA+PMS-40-3	1.279
HGPA+PMS-40-4	4579		HGPA+PMS-40-4	0.552		HGPA+PMS-40-4	1.989		HGPA+PMS-40-4	1.556
HGPA+PMS-40-5	4782		HGPA+PMS-40-5	0.617		HGPA+PMS-40-5	1.986		HGPA+PMS-40-5	1.295
HGPA+PMS-40-6	3686		HGPA+PMS-40-6	0.712		HGPA+PMS-40-6	2.093		HGPA+PMS-40-6	1.557
HGPA+Fer-1-1	5522		HGPA+Fer-1-1	0.458		HGPA+Fer-1-1	2.149		HGPA+Fer-1-1	1.381
HGPA+Fer-1-2	5408		HGPA+Fer-1-2	0.657		HGPA+Fer-1-2	2.079		HGPA+Fer-1-2	1.498
HGPA+Fer-1-3	4005		HGPA+Fer-1-3	0.463		HGPA+Fer-1-3	2.088		HGPA+Fer-1-3	1.458
HGPA+Fer-1-4	4061		HGPA+Fer-1-4	0.527		HGPA+Fer-1-4	2.066		HGPA+Fer-1-4	1.379
HGPA+Fer-1-5	4392		HGPA+Fer-1-5	0.664		HGPA+Fer-1-5	1.957		HGPA+Fer-1-5	1.298
HGPA+Fer-1-6	3354		HGPA+Fer-1-6	0.723		HGPA+Fer-1-6	1.941		HGPA+Fer-1-6	1.334
HGPA+PMS-40+RSL-3-1	6729		HGPA+PMS-40+RSL-3-1	0.91		HGPA+PMS-40+RSL-3-1	2.138		HGPA+PMS-40+RSL-3-1	2.588
HGPA+PMS-40+RSL-3-2	7841		HGPA+PMS-40+RSL-3-2	0.859		HGPA+PMS-40+RSL-3-2	2.13		HGPA+PMS-40+RSL-3-2	2.305
HGPA+PMS-40+RSL-3-3	8273		HGPA+PMS-40+RSL-3-3	0.984		HGPA+PMS-40+RSL-3-3	2.314		HGPA+PMS-40+RSL-3-3	2.271
HGPA+PMS-40+RSL-3-4	8093		HGPA+PMS-40+RSL-3-4	0.797		HGPA+PMS-40+RSL-3-4	2.543		HGPA+PMS-40+RSL-3-4	2.408
HGPA+PMS-40+RSL-3-5	8216		HGPA+PMS-40+RSL-3-5	0.647		HGPA+PMS-40+RSL-3-5	2.213		HGPA+PMS-40+RSL-3-5	2.135
HGPA+PMS-40+RSL-3-6	7366		HGPA+PMS-40+RSL-3-6	0.636		HGPA+PMS-40+RSL-3-6	2.223		HGPA+PMS-40+RSL-3-6	2.516
**Figure6 f-i**										
		**1**			**2**			**3**		
	**Actb**	ΔCT								
	Control	17.322	17.327	17.279	17.296	17.319	17.272	17.347	17.340	17.348
	HGPA	17.330	17.334	17.336	17.306	17.332	17.307	17.336	17.303	17.314
	HGPA+PMS-40	17.329	17.329	17.347	17.326	17.331	17.330	17.309	17.316	17.303
	HGPA+Fer-1	17.300	17.306	17.340	17.295	17.328	17.344	17.325	17.355	17.317
	HGPA+PMS-40+RSL-3	17.347	17.356	17.309	17.310	17.324	17.366	17.346	17.324	17.323
	**Acsl4**	ΔCT								
	Control	23.923	23.31	23.311	23.834	23.499	23.446	23.492	23.609	23.947
	HGPA	22.34	22.343	22.338	22.304	22.32	22.367	22.215	22.201	22.201
	HGPA+PMS-4	22.823	22.829	22.831	22.793	22.826	22.798	22.927	22.325	22.967
	HGPA+Fer-1	22.899	22.929	23.232	23.234	21.925	22.918	23.138	23.196	23.191
	HGPA+PMS-4	22.328	22.349	22.344	22.367	22.315	22.34	22.217	22.232	22.231
	**Ftl1**									
	Control	21.564	21.549	21.533	21.356	21.409	21.341	21.655	21.662	21.648
	HGPA	23.273	23.23	23.224	23.218	23.21	23.271	23.381	23.385	23.317
	HGPA+PMS-4	22.444	22.439	22.422	22.446	22.427	22.443	22.642	22.621	22.641

	HGPA+Fer-1	22.532	22.517	22.537	22.287	22.327	22.329	22.009	22.137	22.729
	HGPA+PMS-4	23.277	23.282	23.306	23.432	23.416	23.498	23.313	23.314	23.272
	**Trf**									
	Control	23.665	23.333	23.313	23.791	23.437	23.437	23.71	23.343	23.349
	HGPA	22.229	22.256	22.298	22.345	22.295	22.337	22.331	22.332	22.341
	HGPA+PMS-4	22.824	23.842	22.195	22.815	23.143	22.866	22.986	22.398	22.909
	HGPA+Fer-1	22.95	23.214	22.932	23.352	22.639	23.018	23.334	22.937	23.534
	HGPA+PMS-4	21.832	22.011	22.35	22.293	22.335	22.015	22.267	21.835	22.102
	**Steap3**									
	Control	23.319	23.3	23.33	23.517	23.523	23.513	23.332	23.351	23.299
	HGPA	22.271	22.331	22.304	22.419	22.423	22.402	22.302	22.335	22.309
	HGPA+PMS-4	22.839	22.837	22.835	22.826	22.852	22.843	22.934	22.926	22.928
	HGPA+Fer-1	22.908	22.957	22.926	23.472	23.415	23.452	22.935	22.916	22.929
	HGPA+PMS-4	22.312	22.356	22.331	22.347	22.376	22.317	22.239	22.219	22.203
**Figure6 k-n**										
	SLC7A11	β-actin			SLC7A11	β-actin			SLC7A11	β-actin
Control	80150	61685		Control	84226	61679		Control	90141	61731
HGPA	12031	63986		HGPA	16049	63975		HGPA	24349	63978
HGPA+PMS-40	74140	57818		HGPA+PMS-40	69699	57817		HGPA+PMS-40	75630	57835
HGPA+Fer-1	69020	53395		HGPA+Fer-1	60712	53396		HGPA+Fer-1	73787	53395
HGPA+PMS-40+RSL-3	13654	58282		HGPA+PMS-40+RSL-3	15672	58277		HGPA+PMS-40+RSL-3	21965	58278
	SLC3A2	β-actin			SLC3A2	β-actin			SLC3A2	β-actin
Control	121758	61685		Control	97981	61679		Control	100321	61731
HGPA	20153	63986		HGPA	12987	63975		HGPA	25252	63978
HGPA+PMS-40	69661	57818		HGPA+PMS-40	63218	57817		HGPA+PMS-40	73824	57835
HGPA+Fer-1	55327	53395		HGPA+Fer-1	49584	53396		HGPA+Fer-1	59552	53395
HGPA+PMS-40+RSL-3	16392	58282		HGPA+PMS-40+RSL-3	8013	58277		HGPA+PMS-40+RSL-3	10041	58278
	GPX4	β-actin			GPX4	β-actin			GPX4	β-actin
Control	175369	61685		Control	188942	61679		Control	184396	61731
HGPA	105083	63986		HGPA	83516	63975		HGPA	99997	63978
HGPA+PMS-40	149810	57818		HGPA+PMS-40	161335	57817		HGPA+PMS-40	156933	57835
HGPA+Fer-1	143114	53395		HGPA+Fer-1	154892	53396		HGPA+Fer-1	152064	53395
HGPA+PMS-40+RSL-3	94159	58282		HGPA+PMS-40+RSL-3	106596	58277		HGPA+PMS-40+RSL-3	103219	58278
**Figure7 a-c**										
T-GSH	A1	A2	GSSG	A1	A2	GSH		GSH/GSSG		
	0.052	0.244		0.149	0.368	Control1	14.63243768	Control1	9.689087002	
	0.051	0.229		0.154	0.383	Control2	8.77201226	Control2	7.972843717	
	0.052	0.237		0.155	0.372	Control3	9.305323075	Control3	6.691822927	
	0.052	0.236		0.148	0.373	Control4	6.341191701	Control4	5.307254871	
Control1	0.382	1.867	Control1	0.188	0.341	Control5	5.707565767	Control5	3.339860656	
Control2	0.39	1.599	Control2	0.192	0.338	Control6	10.40882965	Control6	13.66870806	
Control3	0.341	1.604	Control3	0.203	0.378	HGPA1	2.301153767	HGPA1	1.29114367	
Control4	0.32	1.309	Control4	0.186	0.349	HGPA2	2.065385288	HGPA2	1.041444004	
Control5	0.337	1.41	Control5	0.171	0.413	HGPA3	0.906097509	HGPA3	0.438555073	
Control6	0.421	1.683	Control6	0.225	0.322	HGPA4	1.002360738	HGPA4	0.441883253	
HGPA1	0.396	1.03	HGPA1	0.175	0.407	HGPA5	0.677708285	HGPA5	0.368516013	
HGPA2	0.461	1.057	HGPA2	0.176	0.412	HGPA6	0.102423404	HGPA6	0.064566016	
HGPA3	0.412	0.983	HGPA3	0.202	0.484	HGPA+PMS-4	4.412096339	HGPA+PMS-4	2.738645868	
HGPA4	0.389	0.977	HGPA4	0.161	0.451	HGPA+PMS-4	6.202618946	HGPA+PMS-4	4.913406811	
HGPA5	0.454	0.926	HGPA5	0.201	0.441	HGPA+PMS-4	4.411266163	HGPA+PMS-4	3.747611678	
HGPA6	0.372	0.792	HGPA6	0.18	0.425	HGPA+PMS-4	6.547910877	HGPA+PMS-4	3.93801684	
HGPA+PMS-40-1	0.453	1.236	HGPA+PMS-40-1	0.157	0.356	HGPA+PMS-4	4.942424131	HGPA+PMS-4	2.604791849	
HGPA+PMS-40-2	0.366	1.118	HGPA+PMS-40-2	0.168	0.299	HGPA+PMS-4	2.510739711	HGPA+PMS-4	1.659534689	
HGPA+PMS-40-3	0.415	1.212	HGPA+PMS-40-3	0.141	0.308	HGPA+Fer-1-1	6.617933915	HGPA+Fer-1-1	4.327512512	
HGPA+PMS-40-4	0.387	1.452	HGPA+PMS-40-4	0.159	0.375	HGPA+Fer-1-2	3.673451209	HGPA+Fer-1-2	3.203736803	
HGPA+PMS-40-5	0.415	1.26	HGPA+PMS-40-5	0.185	0.406	HGPA+Fer-1-3	8.128602306	HGPA+Fer-1-3	6.602358399	
HGPA+PMS-40-6	0.506	1.135	HGPA+PMS-40-6	0.209	0.416	HGPA+Fer-1-4	7.903732342	HGPA+Fer-1-4	4.912353406	
HGPA+Fer-1-1	0.414	1.407	HGPA+Fer-1-1	0.185	0.374	HGPA+Fer-1-5	3.325544984	HGPA+Fer-1-5	3.606072157	
HGPA+Fer-1-2	0.384	1.192	HGPA+Fer-1-2	0.157	0.344	HGPA+Fer-1-6	5.61670284	HGPA+Fer-1-6	3.86196069	
HGPA+Fer-1-3	0.386	1.636	HGPA+Fer-1-3	0.152	0.327	HGPA+PMS-4	-0.322008305	HGPA+PMS-4	-0.151614991	

HGPA+Fer-1-4	0.389	1.491	HGPA+Fer-1-4	0.175	0.367	HGPA+PMS-4	4.264482481	HGPA+PMS-4	2.408511378	
HGPA+Fer-1-5	0.435	1.096	HGPA+Fer-1-5	0.16	0.302	HGPA+PMS-4	0.764504947	HGPA+PMS-4	0.43733488	
HGPA+Fer-1-6	0.453	1.334	HGPA+Fer-1-6	0.202	0.383	HGPA+PMS-4	0.931704309	HGPA+PMS-4	0.458091814	
HGPA+PMS-40+RSL-3-1	0.38	0.733	HGPA+PMS-40+RSL-3-1	0.157	0.387	HGPA+PMS-4	1.299054311	HGPA+PMS-4	0.565982762	
HGPA+PMS-40+RSL-3-2	0.483	1.281	HGPA+PMS-40+RSL-3-2	0.18	0.398	HGPA+PMS-4	1.391536902	HGPA+PMS-4	0.891834734	
HGPA+PMS-40+RSL-3-3	0.414	0.839	HGPA+PMS-40+RSL-3-3	0.205	0.415					
HGPA+PMS-40+RSL-3-4	0.335	0.882	HGPA+PMS-40+RSL-3-4	0.181	0.449					
HGPA+PMS-40+RSL-3-5	0.42	0.893	HGPA+PMS-40+RSL-3-5	0.199	0.421					
HGPA+PMS-40+RSL-3-6	0.419	0.82	HGPA+PMS-40+RSL-3-6	0.179	0.346					
					**Figure7 d-f**					
		**1**			**2**			**3**		
	**Actb**									
	Control1	17.332	17.320	17.310	17.292	17.319	17.332	17.344	17.333	17.361
	Control2	17.321	17.331	17.302	17.343	17.343	17.349	17.293	17.330	17.318
	Control3	17.294	17.305	17.355	17.324	17.312	17.301	17.343	17.316	17.329
	Control4	17.301	17.323	17.320	17.305	17.296	17.287	17.364	17.337	17.330
	Control5	17.331	17.352	17.322	17.312	17.357	17.308	17.372	17.336	17.323
	**Slc3a2**									
	Control1	21.36	21.36	21.361	21.308	21.387	21.41	21.755	21.762	21.758
	Control2	23.271	23.227	23.225	23.118	23.159	23.138	23.269	23.235	23.243
	Control3	22.609	22.647	22.631	22.423	22.429	22.43	22.405	22.473	22.403
	Control4	22.304	22.326	22.3	22.412	22.425	22.428	22.312	22.304	22.278
	Control5	23.285	23.327	23.271	23.315	23.296	23.323	23.132	23.187	23.191
	**Slc7a11**									
	Control1	21.38	21.321	21.346	21.332	21.338	21.342	21.735	21.754	21.781
	Control2	23.268	23.207	23.228	23.245	23.23	23.234	23.362	23.337	23.348
	Control3	22.39	22.413	22.461	22.505	22.045	22.539	22.433	22.422	22.406
	Control4	22.312	22.327	22.297	22.343	22.283	22.295	22.268	22.222	22.219
	Control5	23.297	23.335	23.36	23.165	23.183	23.189	23.293	23.288	23.319
	**Gpx4**									
	Control1	21.343	21.368	21.362	21.631	21.666	21.625	21.868	21.859	21.864
	Control2	23.328	23.335	23.338	23.258	23.23	23.238	23.256	23.247	23.255
	Control3	22.144	22.536	22.568	22.434	21.412	22.461	22.412	22.429	22.113
	Control4	22.198	22.175	22.185	22.304	22.295	22.316	22.324	22.312	22.349
	Control5	23.3	23.338	23.31	23.32	23.336	23.331	23.174	23.144	23.124
					**Figure7 g-j**					
	ACSL4	β-actin			ACSL4	β-actin			ACSL4	β-actin
Control	25299	128815		Control	23948	134766		Control	28574	130223
HGPA	111367	130369		HGPA	130282	137464		HGPA	128882	135691
HGPA+PMS-40	74888	128956		HGPA+PMS-40	77622	136669		HGPA+PMS-40	68940	143000
HGPA+Fer-1	55330	137125		HGPA+Fer-1	59381	161332		HGPA+Fer-1	69297	143782
HGPA+PMS-40+RSL-3	119812	139906		HGPA+PMS-40+RSL-3	148944	142261		HGPA+PMS-40+RSL-3	151435	143068
	FTL	β-actin			FTL	β-actin			FTL	β-actin
Control	175853	128815		Control	162992	134766		Control	200263	130223
HGPA	80970	130369		HGPA	72505	137464		HGPA	100236	135691
HGPA+PMS-40	139436	128956		HGPA+PMS-40	130411	136669		HGPA+PMS-40	157928	143000
HGPA+Fer-1	150766	137125		HGPA+Fer-1	140104	161332		HGPA+Fer-1	177795	143782
HGPA+PMS-40+RSL-3	98496	139906		HGPA+PMS-40+RSL-3	78727	142261		HGPA+PMS-40+RSL-3	85825	143068
	Transferrin	β-actin			Transferrin	β-actin			Transferrin	β-actin
Control	24537	128815		Control	27724	134766		Control	20542	130223
HGPA	81377	130369		HGPA	79635	137464		HGPA	71380	135691
HGPA+PMS-40	45973	128956		HGPA+PMS-40	44765	136669		HGPA+PMS-40	50304	143000
HGPA+Fer-1	44317	137125		HGPA+Fer-1	43218	161332		HGPA+Fer-1	43522	143782
HGPA+PMS-40+RSL-3	92015	139906		HGPA+PMS-40+RSL-3	95444	142261		HGPA+PMS-40+RSL-3	84211	143068
	steap3	β-actin			steap3	β-actin			steap3	β-actin
Control	82001	128815		Control	57574	134766		Control	96307	130223
HGPA	141926	130369		HGPA	141868	137464		HGPA	163667	135691

HGPA+PMS-40	96102	128956		HGPA+PMS-40	95870	136669		HGPA+PMS-40	122418	143000
HGPA+Fer-1	89024	137125		HGPA+Fer-1	93647	161332		HGPA+Fer-1	99238	143782
HGPA+PMS-40+RSL-3	201123	139906		HGPA+PMS-40+RSL-3	147805	142261		HGPA+PMS-40+RSL-3	146987	143068
