# Supplementary material for: Mechanism of Plantamajoside in inhibiting ferroptosis of pancreatic β cells and treatment of T2DM via activation of the xCT/GPX4 pathway
Source: PLoS One. 2025 Jun 20;20(6):e0325674. doi: 10.1371/journal.pone.0325674 (PMC12180730; doi:10.1371/journal.pone.0325674)
Supplement: S1 Table — (DOCX) [file pone.0325674.s002.docx]

**1 Reagents**

Plantamajoside (B20225) and Metformin (B25331) were purchased from Shanghai yuanye Bio-Technology Co., Ltd. Ferrostatin-1 (S81461) were purchased from Shanghai yuanye Bio-Technology Co., Ltd. 1S,3R-RSL 3 (R873890) was purchased from Shanghai Macklin Biochemical Technology Co., Ltd. Glucose (G8150) was purchased from Solarbio. Palmitic acid (P9767) was purchased from Sigma. Elisa kit for insulin (ml001983) was purchased from Shanghai Enzyme-linked Biotechnology Co., Ltd. TUNEL Apoptosis Assay Kit (KGA1406) was purchased from Jiangsu Kaiji Bio-technology Co., Ltd. Perls Prussian blue staining kit (G1428) was purchased from Solarbio.Methane dicarboxylic aldehyde (A003-1-2), reactive oxygen species (E004-1-1), total iron (A039-2-1), total GSH/GSSG (A061-1-2) were obtained from Nanjing Jiancheng Biological Engineering Institute. Elisa kits for 4-hydroxynonenal (EK-M25633) was purchased from Shanghai Enzyme Research Biotechnology Co., Ltd. Primary antibodies for SLC7A11 (26864-1-AP), SLC3A2 (15193-1-AP), GPX4 (30388-1-AP), Transferrin (17435-1-AP), Steap3 (28478-1-AP), FTL (10727-1-AP) and Beta Actin (20536-1-AP) were purchased from Proteintech Group, Inc. Primary antibodies for ASCL4 (PA5-89830) was purchased from Thermo Fisher Scientific Inc.

**Table S1 Primer sequence**

| **Genes** | **Primer sequence (5’-3’)** |
| --- | --- |
| ***Actb*** | Forward: CCCCTGAACCCTAAGGCCA |
|  | Reverse: ATGGCTACGTACATGGCTGG |
| ***Slc7a11*** | Forward: GGTCAGAAAGCCAGTTGTGG |
|  | Reverse: AGTATGCCCTTGGGGGAGAT |
| ***Slc3a2*** | Forward: CTCTCTGTTGCACGGTGACT |
|  | Reverse: TTATGCCAGCAGGGAGGTTG |
| ***Gpx4*** | Forward: CCGTCTGAGCCGCTTACTTA |
|  | Reverse: GTGACGATGCACACGAAACC |
| ***Ascl4c*** | Forward: CACTGTCCCCTCAGACACAC |
|  | Reverse: TCAGCCCATATCCCTGACCA |
| ***Trf*** | Forward: GGCATCGGACACTAGCATCA |
|  | Reverse: GCGCAGCCTTGACTGAAAAA |
| ***Steap3*** | Forward: GATGCCCTTGTCTGTGGTCA |
|  | Reverse: GCATCGCGAAGAAGAAGCTG |
| ***Ftl*** | Forward: TTCCTGGAAAGCCACTATCTG |
|  | Reverse: GAGTGAGGCGCTCAAAGAGAT |
