# Supplementary material for: Mechanism of Plantamajoside in inhibiting ferroptosis of pancreatic β cells and treatment of T2DM via activation of the xCT/GPX4 pathway
Source: PLoS One. 2025 Jun 20;20(6):e0325674. doi: 10.1371/journal.pone.0325674 (PMC12180730; doi:10.1371/journal.pone.0325674)
Supplement: S2 Data — (PDF) [file pone.0325674.s003.pdf]

Figure 2 m

ACSL4 79 kDa

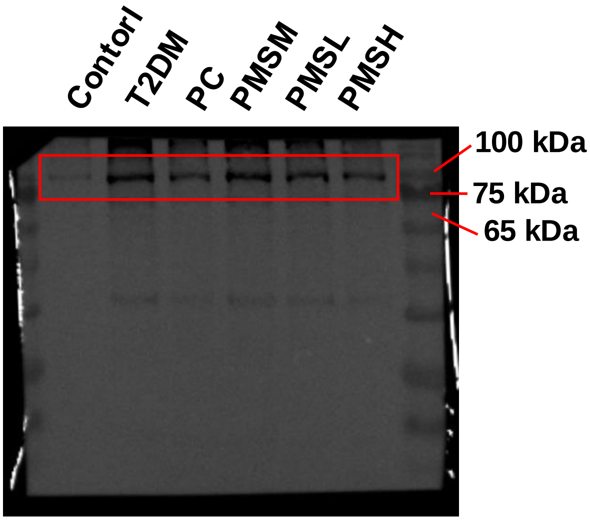

FTL 19 kDa

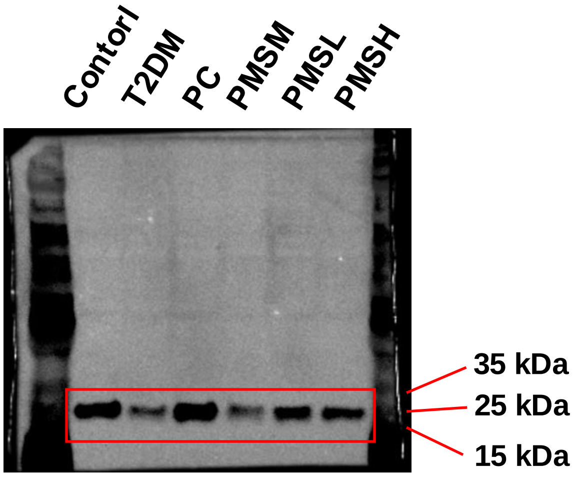

TRF 77 kDa

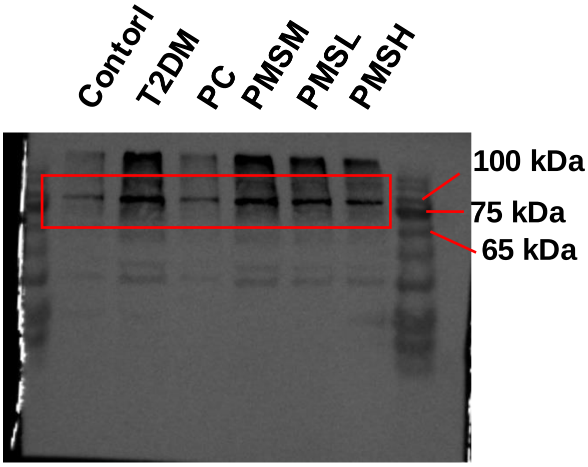

STEAP3 56 kDa

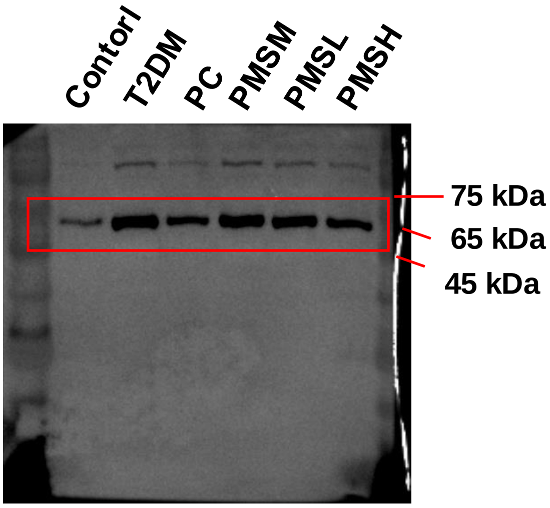

β-actin 42 kDa

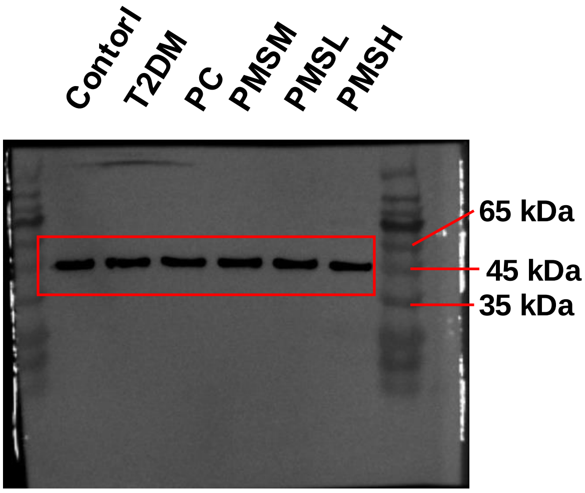

Figure 3 g

SLC7A11 55 kDa

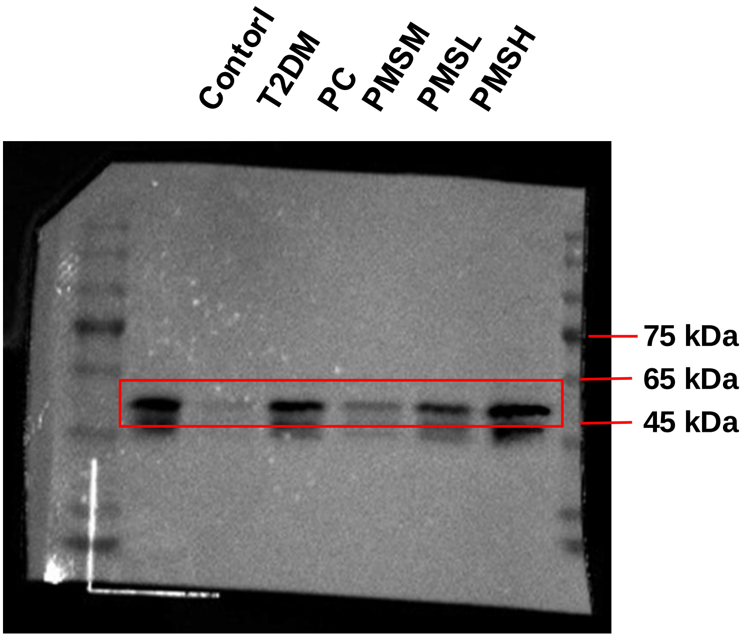

SLC3A2 120 kDa

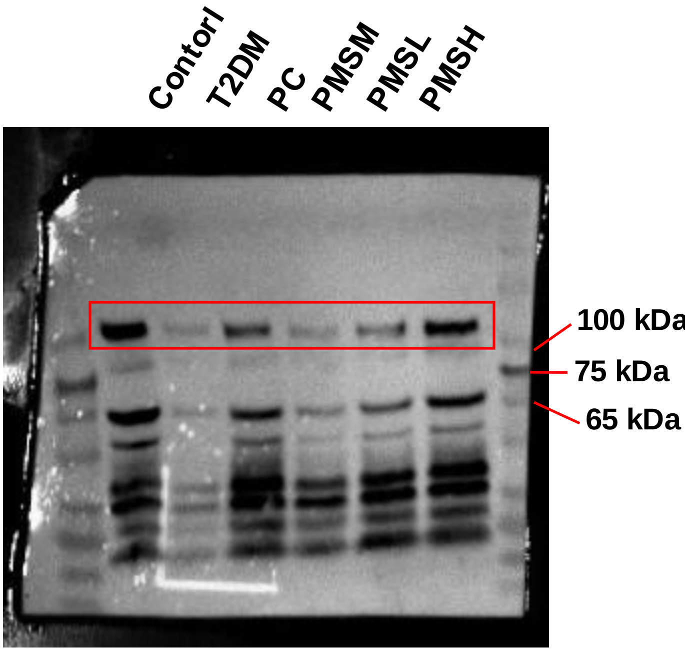

GPX4 23 kDa

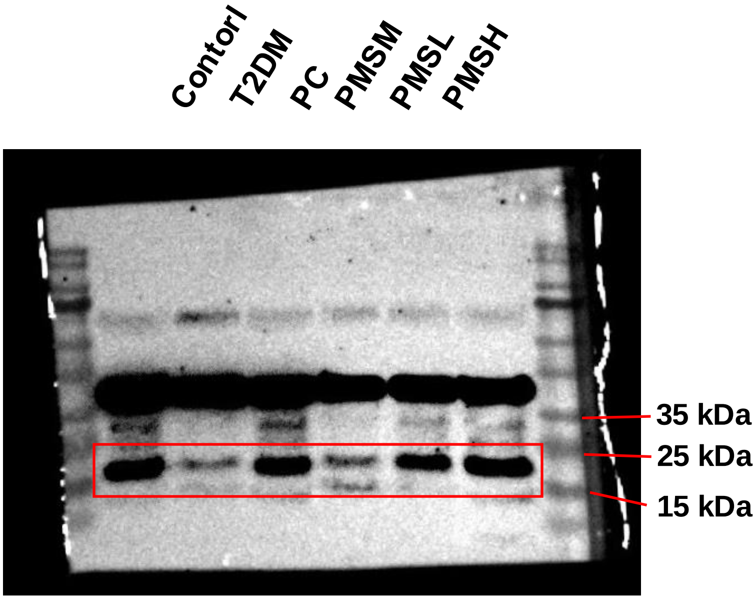

β-actin 42 kDa

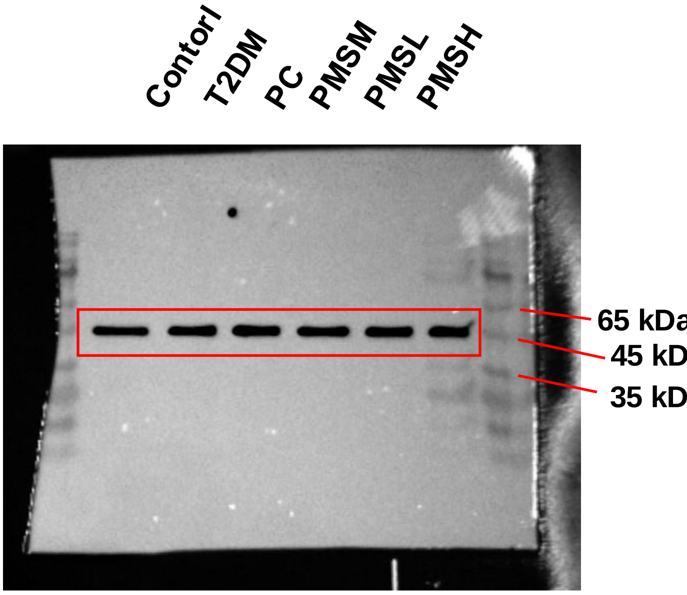

Figure 4 k

ACSL4 79 kDa

HGPA - + +
PMS (μM) - - 40

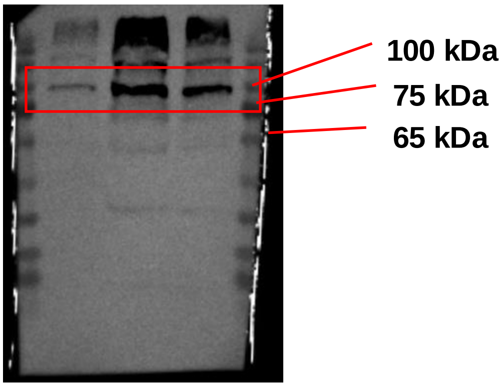

FTL 19 kDa

HGPA - + +
PMS (μM) - - 40

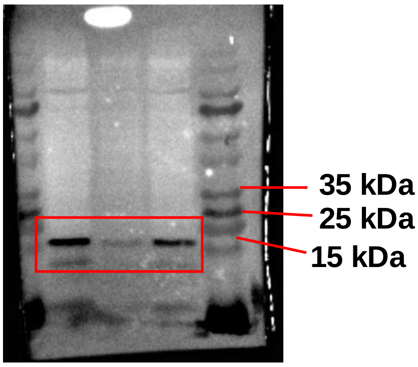

TRF 77 kDa

HGPA - + +
PMS (μM) - - 40

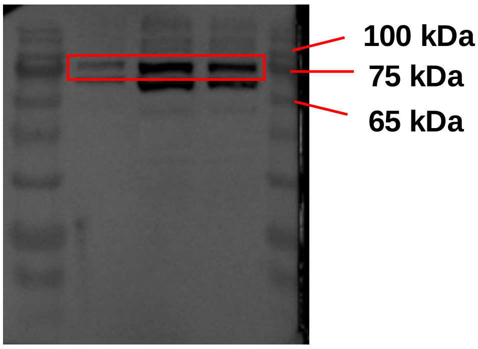

STEAP3 56 kDa

HGPA - + +
PMS (μM) - - 40

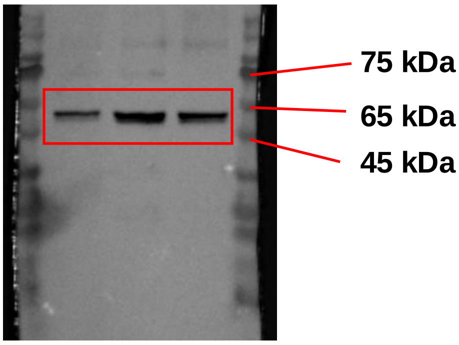

β-actin 42 kDa

HGPA - + +
PMS (μM) - - 40

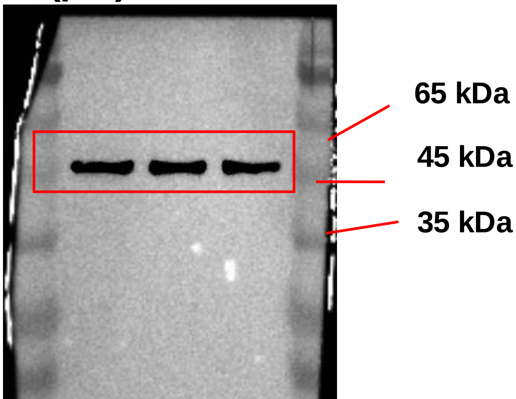

Figure 5 g

SLC7A11 55 kDa

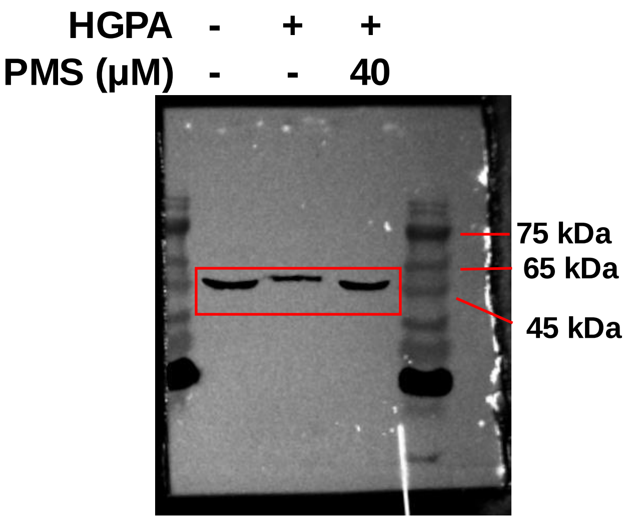

SLC3A2 120 kDa

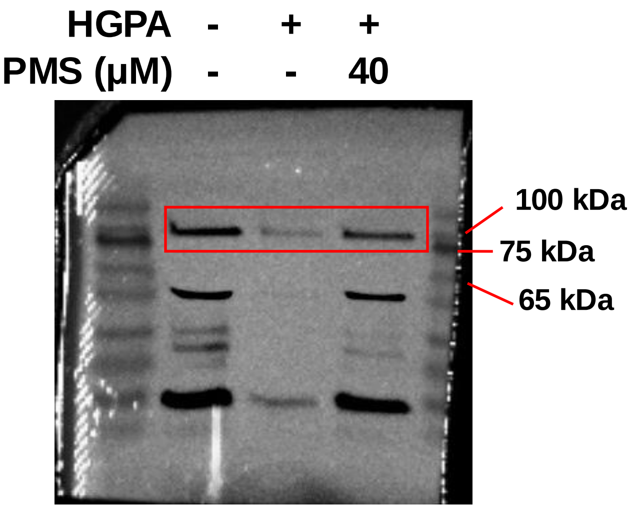

GPX4 23 kDa

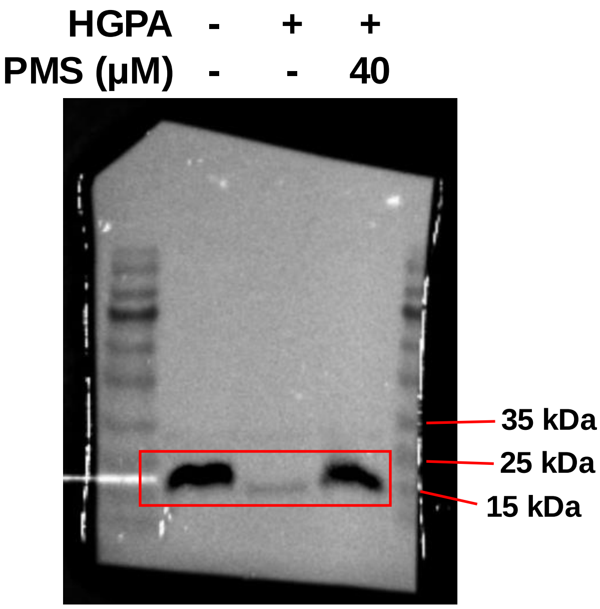

β-actin 42 kDa

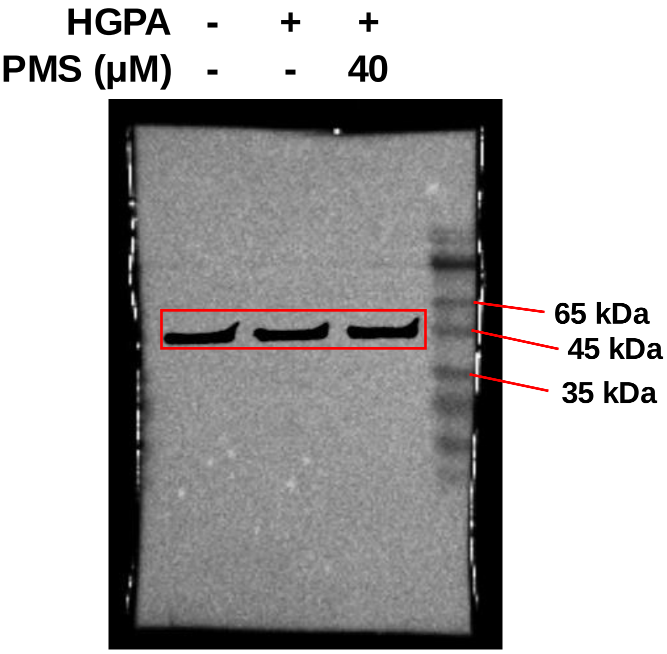

Figure 6 j

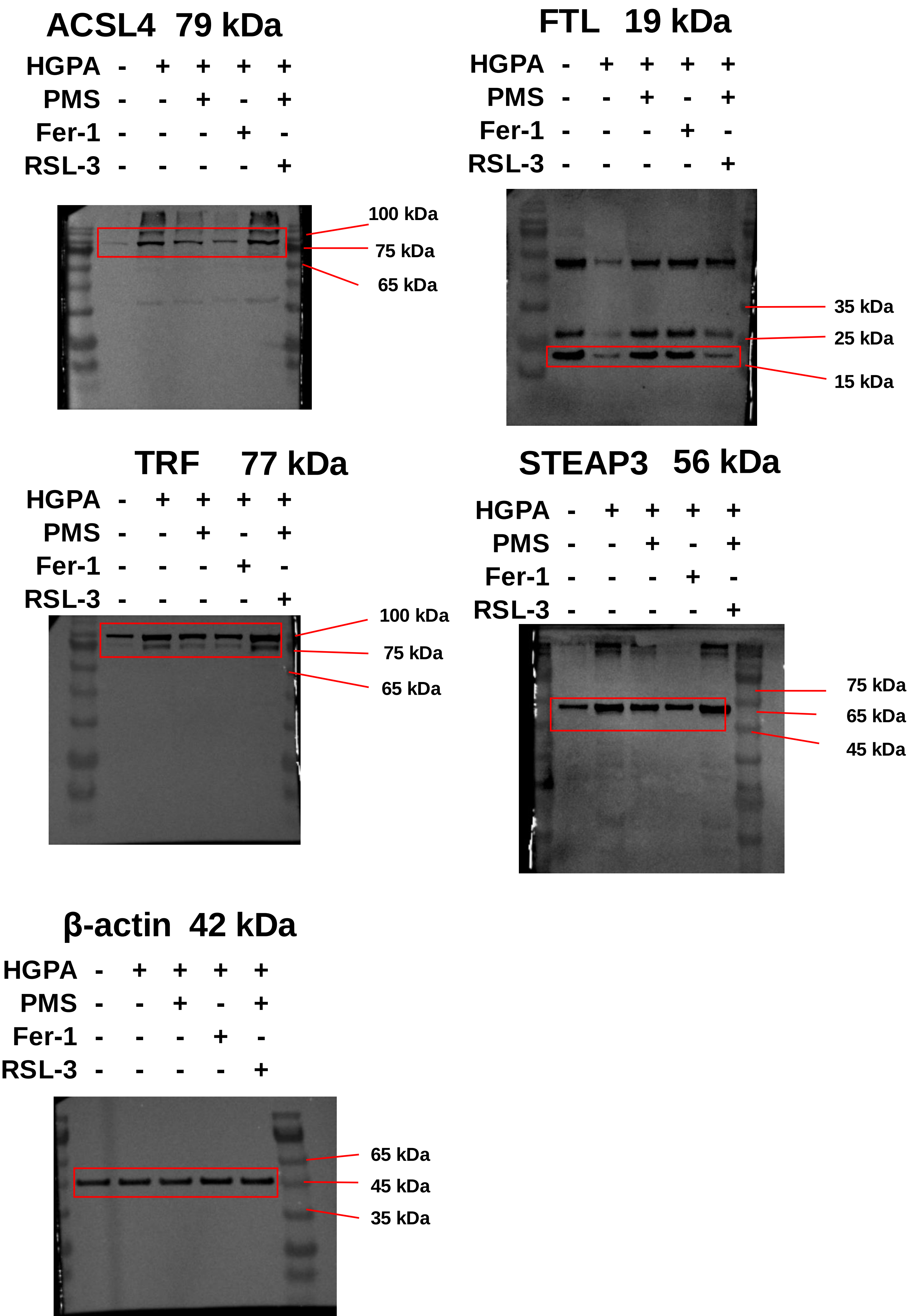
ACSL4 79 kDa
HGPA - + + + +
PMS - - + - +
Fer-1 - - - + -
RSL-3 - - - - +
100 kDa
75 kDa
65 kDa
FTL 19 kDa
HGPA - + + + +
PMS - - + - +
Fer-1 - - - + -
RSL-3 - - - - +
35 kDa
25 kDa
15 kDa
TRF 77 kDa
HGPA - + + + +
PMS - - + - +
Fer-1 - - - + -
RSL-3 - - - - +
100 kDa
75 kDa
65 kDa
STEAP3 56 kDa
HGPA - + + + +
PMS - - + - +
Fer-1 - - - + -
RSL-3 - - - - +
75 kDa
65 kDa
45 kDa
β-actin 42 kDa
HGPA - + + + +
PMS - - + - +
Fer-1 - - - + -
RSL-3 - - - - +
65 kDa
45 kDa
35 kDa

Figure 7 g

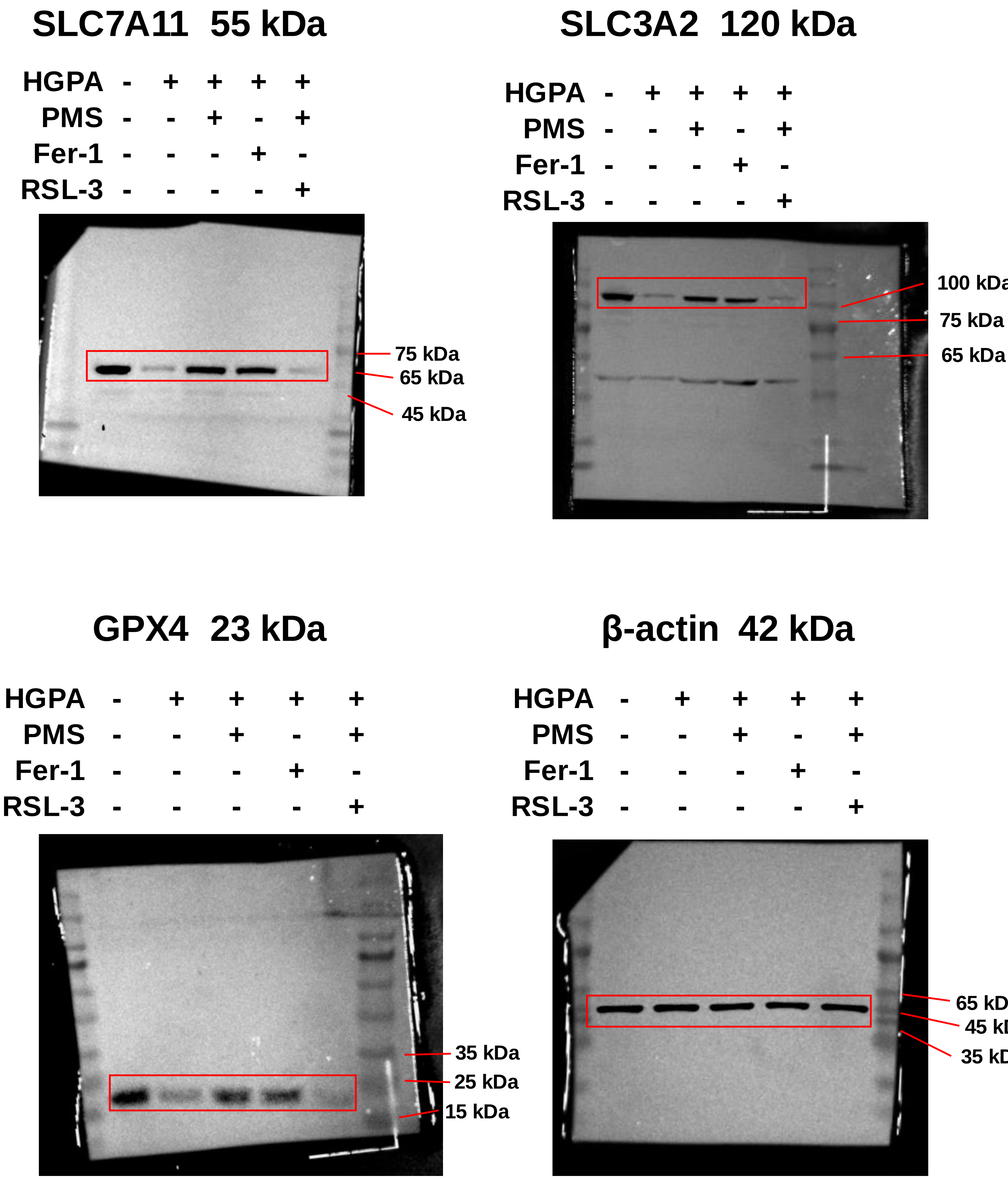
SLC7A11 55 kDa
HGPA - + + + +
PMS - - + - +
Fer-1 - - - + -
RSL-3 - - - - +
75 kDa
65 kDa
45 kDa
SLC3A2 120 kDa
HGPA - + + + +
PMS - - + - +
Fer-1 - - - + -
RSL-3 - - - - +
100 kD
75 kDa
65 kDa
GPX4 23 kDa
HGPA - + + + +
PMS - - + - +
Fer-1 - - - + -
RSL-3 - - - - +
35 kDa
25 kDa
15 kDa
β-actin 42 kDa
HGPA - + + + +
PMS - - + - +
Fer-1 - - - + -
RSL-3 - - - - +
65 kD
45 k
35 k
