# Supplementary material for: Mechanism of Plantamajoside in inhibiting ferroptosis of pancreatic β cells and treatment of T2DM via activation of the xCT/GPX4 pathway
Source: PLoS One. 2025 Jun 20;20(6):e0325674. doi: 10.1371/journal.pone.0325674 (PMC12180730; doi:10.1371/journal.pone.0325674)
Supplement: Attachment — Submitted filename: Response_to_Reviewers_auresp_2.docx [file pone.0325674.s005.docx]

**Reviewer #1:**

**Issue 1: Figure 1, the sub-panel label “g” is absent.**

**Respond：We added the sub-panel label “g” in figure 1.**

**Issue 2: Figure 2d, no raw data.**

**Response: We have added the raw data for Figure 2d.**

**Issue 3: The raw data sheet “Figure2 i—I” should be corrected to “Figure2 i—l”.**

**Response: We have corrected “Figure2 i—I” to “Figure2 i—l”.**

**Issue 4: Many histograms/panels have been changed with different data or numbers. Please clarify or explain these changes clearly. For example, in figure 2n, all the numbers appear to have changed. The most obvious changes are in the PC and control group. The PC group has a level greater than 0.5 with a p<0.05, whereas in the original version, this number was less than 0.5 with a p<0.01. Similarly, the control group also shows a higher value compared to the original version. What are the reasons for these changes?**

**Response:** **Thank you for your attention to detail regarding the changes in the histograms and data panels.** **When uploading the original data, we carefully checked and re-analyzed all the data, which led to some differences from the initial version.**

**Issue 5: Similar issues occur for other panels in other figures such as figure 2l (ftl1 mRNA) PC group, figure 2o (trf protein) all groups, figure 2p (steap3 protein) all groups, figure 2q (ftl protein) all groups, and figure 3j (gpx4 protein) PC group. Please clarify or explain these changes clearly.**

**Response:** **Thank you for your attention to detail regarding the changes in the histograms and data panels.** **When uploading the original data, we carefully checked and re-analyzed all the data, which led to some differences from the initial version.**

**Issue 6: Several histograms/panels have updated different numbers and P value labels in Figures 4-7, compared to the original version. Please clarify or explain these changes clearly for each sub-figure that has changed.**

**Response:** **Thank you for your attention to detail regarding the changes in the histograms and data panels.** **When uploading the original data, we carefully checked and re-analyzed all the data, which led to some differences from the initial version.**

**Issue 7: Figure 4K, the bands in the TRF raw image do not match the bands in figure 4K. Please also update the raw images to correspond to the TRF bands in figure 4K.**

**Response: We have updated the correct original images to avoid changes caused by incorrect stretching.
Issue 8: Similar issues occur for figure 5g; all the bands do not match the raw images well, especially the SLC7A11 and beta-actin raw images. Did the authors mistakenly shorten the images horizontally? All the bands appear strange compared to the raw images. Please also update the images correctly.**

**Response: We have updated the correct original images to avoid changes caused by incorrect stretching.**
